# Development and Evaluation of a BCG/BCP-Based Cellulose Acetate Freshness Indicator for Beef Loin During Cold Storage

**DOI:** 10.3390/foods14234017

**Published:** 2025-11-23

**Authors:** Kyung-Jik Lim, Jun-Seo Kim, Yu-Jin Heo, Han-Seung Shin

**Affiliations:** Department of Food Science and Biotechnology, Dongguk University-Seoul, 32, Dongguk-ro, Ilsandong-gu, Goyang-si 410-820, Gyeonggi-do, Republic of Korea; kyung9209@naver.com (K.-J.L.); khkjs95@naver.com (J.-S.K.); pdp0616@naver.com (Y.-J.H.)

**Keywords:** freshness indicator (FI), cellulose acetate (CA), bromocresol dyes, beef quality, volatile total basic-nitrogen (TVB-N)

## Abstract

Monitoring the freshness of perishable foods remains a challenge due to the lack of simple and reliable indicators that can visually reflect chemical and microbial changes. In this study, a colorimetric freshness indicator was developed using bromocresol green (BCG) and bromocresol purple (BCP), two pH-sensitive dyes with complementary transition ranges, to provide a visible and quantitative response corresponding to beef quality during cold storage. Cellulose acetate (CA) films were prepared by incorporating the dyes with different plasticizers—glycerol and polyethylene glycol (PEG 200 and PEG 400)—at varying ratios, resulting in 24 formulations. Based on color stability and sensitivity to trimethylamine (TMA) vapor, two optimized indicators were selected for further packaging tests with beef samples stored at 4 °C. Beef freshness was evaluated by total bacterial count (TBC), total volatile basic nitrogen (TVB-N), and pH, while volatile amines in the headspace were quantified using solid-phase microextraction–gas chromatography–flame ionization detection (SPME–GC–FID). The color difference (ΔE) of the indicators showed strong correlations with TBC and TVB-N, and a threshold of ΔE ≈ 12 provided a practical visual cue corresponding to the microbiological safety limit. The two indicators exhibited complementary functions, with G100-1 acting as an early-warning sensor and G100-2 maintaining contrast at later stages. These findings demonstrate the potential of this dual-indicator system as a simple, non-destructive tool for intelligent packaging applications.

## 1. Introduction

Traditional food packaging has primarily served to protect products from environmental factors such as heat, light, moisture, and microbial contamination [1]. Nowadays, packaging is expected to play a more active role, not only in maintaining food quality but also in reducing waste and ensuring the safe delivery of products to consumers [2]. As global concerns over food safety and sustainability increase, packaging that provides real-time quality information has become a priority. Intelligent packaging systems have therefore attracted significant attention as tools to monitor the condition of packaged foods [3]. These systems include freshness indicators, time–temperature indicators, and gas indicators [4]. Freshness indicators are particularly attractive because visible color changes can be observed in response to the volatile compounds, metabolites—such as volatile basic nitrogen (VBN)—and other detectable markers generated by microbial growth or metabolism during the storage of different foods [5]. Among these, colorimetric freshness indicators have gained increasing attention because they can be easily integrated into multilayer film structures and provide direct, real-time feedback on product quality.

A typical freshness indicator consists of a matrix including a polymer, substrate, and lamination layer with a colorant, and may also contain a plasticizer to improve film flexibility [6,7,8].

Cellulose acetate (CA) was selected as the polymer matrix because of its transparency, biocompatibility, non-toxicity, and biodegradability. These characteristics make it an ideal support for dye immobilization in food packaging applications. To improve the mechanical flexibility and physicochemical stability of CA-based films, plasticizers were incorporated into the formulation [9]. In this study, glycerol and polyethylene glycols (PEG 200 and PEG 400) were chosen as plasticizers to enhance film workability and maintain uniform dye dispersion. Low-molecular-weight polyethylene glycols (PEG 200–400) are known for their excellent chemical stability, biocompatibility, and non-toxicity [10]. Such properties make them particularly suitable for food and pharmaceutical use. PEG-400 has been reported to increase the hydrophobicity and crystallinity of polymer membranes, thereby improving their resistance to water vapor and enhancing gas permeability [11]. In contrast, glycerol-containing films exhibit remarkable color responsiveness within the pH range typical of food spoilage. The combination of CA and these plasticizers yields a stable and flexible polymeric support that effectively preserves the activity of pH-sensitive dyes in intelligent freshness indicators [10]. For the sensing system, a mixture of bromocresol green (BCG, pKa ≈ 4.7) and bromocresol purple (BCP, pKa ≈ 6.3) was employed. This combination broadens the effective pH response range to approximately 4.5–6.5 [12]. The color transition of bromocresol dyes originates from a reversible ionization equilibrium between their acidic (HIn) and basic (In^−^) forms, as described by the Henderson–Hasselbalch relationship. As volatile amines increase the local pH during beef spoilage, this equilibrium shifts toward the deprotonated form, resulting in a visible color transition.

This range corresponds to the gradual pH increase observed in beef during chilled storage (from pH 5.6 to 6.2) [13]. It enables sensitive detection of subtle pH variations in real food systems. Both indicators are thermally and biologically stable, showing no toxicity during sterilization [12]. Accordingly, this dye mixture was selected to ensure clear, reversible, and visually distinct color differentiation in response to volatile basic nitrogen compounds in the headspace of beef packaging. In this study, the developed indicator changes color in response to variations in pH.

During the spoilage of fresh meat, trimethylamine oxide (TMAO) is decomposed into ammonia gas and trimethylamine (TMA). These volatile basic nitrogen compounds and volatile amines are key indicators used to assess meat freshness. Volatile amines such as ammonia, dimethylamine (DMA), and TMA are produced through amino acid degradation and the microbial activity [14]. This is attributed to the activity of psychrotrophic bacteria under aerobic conditions, which generate ammonia through the deamination of amino acids. Moreover, several microorganisms, including *Pseudomonas*, *Photobacterium*, and *Vibrionaceae*, have been reported to contribute to the accumulation of TVB-N by producing ammonia and methylamines, showing a general correlation between microbial growth and TVB-N increase during beef storage. Therefore, the concentration of TVB-N serves as a crucial indicator of meat spoilage [15]. As these gases accumulate during storage, the pH of the headspace gradually increases, leading to a visible color transition in the freshness indicator (FI). Therefore, this study aims to develop a colorimetric freshness indicator capable of reflecting the pH variation induced by volatile amines during beef spoilage, ultimately allowing consumers to visually assess meat freshness through color change.

Gas chromatography (GC) equipped with a flame ionization detector (FID) was utilized to quantify TMA among volatile amines produced during beef spoilage. GC–FID provides excellent sensitivity, reproducibility, and linearity for quantitative determination of volatile organic compounds, whereas GC—Mass Spectrometry (MS) is more suitable for qualitative identification [16]. Previous studies have confirmed that TMA, together with other TVB-N components, is generated during the degradation of amino acids and microbial activity in beef. Accordingly, a quantitative rather than qualitative analytical approach was adopted. Furthermore, solid-phase microextraction (SPME) coupled with gas chromatography was employed as a sensitive and loss-minimizing method for detecting trace volatile compounds. This technique has been widely reported to prevent analyte loss during headspace sampling of packaged foods [17,18,19,20].

Many studies have reported the development of freshness indicators; however, few have directly compared their performance with the actual composition of the package headspace under realistic cold-chain conditions. This limitation is particularly evident in beef products, where previous studies have often shown only modest color shifts under storage conditions that do not fully represent commercial distribution, thereby restricting their practical relevance [21,22]. To address these limitations, the present study developed BCG/BCP-based freshness indicators plasticized with glycerol, PEG 200 and PEG 400 and evaluated their applicability to chilled beef packaging. Beef quality was analyzed through total bacterial counts (TBC), TVB-N, and pH, while volatile amines in the package headspace were quantified using CAR/PDMS (75 µm) SPME fibers coupled with GC–FID. By correlating label color variations with microbiological and chemical quality parameters, this study demonstrates that pH-responsive labels can serve as practical, non-destructive sensors for monitoring beef freshness in real cold-chain conditions, thereby contributing to improved quality control and food waste reduction.

## 2. Materials and Methods

### 2.1. Chemicals and Reagents

Beef loin (400 g per fillet) was purchased 48 h post-slaughter in the local market (Goyang, Korea) and transferred to the laboratory in an ice box. Then, the beef loin was placed in a polypropylene tray (20 cm × 11 cm × 5 cm) and sealed with polyethylene (PE) film, after which it was stored in the refrigerator at 4 °C for 10 days. All beef loin used in this study was obtained from the same carcass.

Cellulose Acetate (CA, CAS No. 9004-35-7), sodium hydroxide (0.01N, CAS No. 1310-73-2), and sulfuric acid (0.01N, CAS No. 7664-93-9) were purchased from Daejung Chemicals and Metals Co., Ltd. (Siheung, Korea). Bromocresol green (BCG, CAS No. 76-60-8) and bromocresol purple (BCP, CAS No. 115-40-2) were purchased from Samchun (Yongin, Korea). Glycerol (CAS No. 56-81-5), polyethylene glycol 200 (PEG200, CAS No. 25322-68-3), and polyethylene glycol 400 (PEG400, CAS No. 25322-68-3) were purchased from Duksan Pure Chemicals (Seoul, Korea). Filter paper and the dense polytetrafluoroethylene (d-PTFE) membrane filter were purchased from Hyundai Micro Co. Ltd. (Seoul, Korea). Polyethylene terephthalate (PET) film and PE film were purchased from Moapack (Hwaseong, Korea). Methylamine hydrochloride (MA, CAS No. 593-51-1), dimethylamine hydrochloride (DMA, CAS No. 506-59-2), and trimethylamine hydrochloride (TMA, CAS No. 593-81-7) were obtained from Sigma-Aldrich Co. (St. Louis, MO, USA). The 75 µm CAR/PDMS solid-Phase Microextraction (SPME) fiber was purchased from Supelco (Bellefonte, PA, USA).

### 2.2. Fabrication of Freshness Indicator

**Figure 1:**
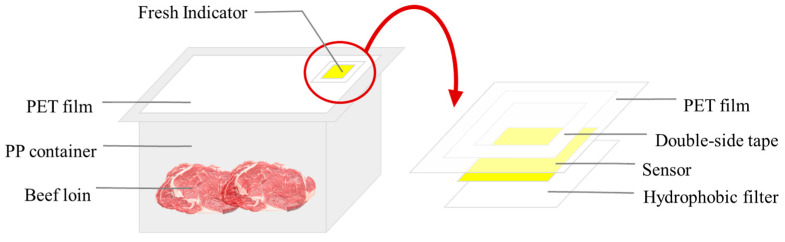
Prototype freshness indicator for beef loin.

The prototype of the developed freshness indicator is shown in Figure 1. The filter paper strip was immersed in the polymer solution (CA) for 10 min. After absorption, the polymer- coated filter paper strip was dried for 3 h in a dark room and cut into squares (2 cm × 2 cm). The freshness indicator was placed on a hydrophobic d-PTFE membrane filter, which was used as a white background and prevented contact with humidity. Then, the PET film was placed on the hydrophobic d-PTFE membrane filter and laminated. Double-sided tape was attached above the PET film for adhesion.

The freshness indicator was fabricated by dipping a filter paper strip in the freshness indicator polymer solution. To prepare the freshness indicator polymer solution, 3 g of CA was fully dissolved in 60 mL of acetone. Then, to provide the optimum concentration of indicator dyes, 60 mg of BCG and BCP mixtures were prepared at different ratios (1:0, 2:1, 1:1, 1:2, and 0:1, *w*/*w*) and added to the CA solution. A series of plasticizers was applied to select the optimum concentration of plasticizer. The plasticizer concentration was 20%, 60%, 100%, and 140% (*w*/*w* of CA). In this study, G, 200P, and 400P denote glycerol, PEG200, and PEG400, respectively. Based on these abbreviations, the indicator films were coded according to the plasticizer type and ratio, and the sample codes with their detailed compositions are summarized in Table 1.

### 2.3. Freshness Indicator Assessment

To evaluate beef quality, the freshness indicator was laminated with PET film and placed above the meat surface inside the package without direct contact. The packaged beef was stored under the same conditions as the beef samples. The color change was captured using a digital camera, and colorimetric analysis was carried out every 2 days using a colorimeter. This setup allowed continuous monitoring of the same indicator film over time while preventing direct contact with the meat surface or moisture, thereby minimizing potential contamination and ensuring consistent color development.

### 2.4. Colorimetric Analysis

The freshness indicator was attached to the inside of a clear glass bottle with a volume of 1.1 L. Then, 10 µL of TMA stock solution was dispensed into the bottle and hermetically sealed with parafilm [23]. The bottle was left at room temperature for 2 h to vaporize the stock solution. The concentration of TMA vapor (mg/kg) was calculated using Equation (1):

(1) $$C_{V}=\frac{V_{L}\;\times \;WF\;\times \;D\times \;24.45}{MW\times \;V_{V}}$$

where *C_V_* is the concentration of TMA vapor in the glass bottle, *V_L_* is the volume of TMA stock solution, *WF* is the mass fraction of TMA stock solution, *D* is the density of TMA stock solution, *MW* is the molecular weight of TMA, and *V_V_* is the volume of the glass bottle.

After 2 h, the color of the freshness indicator was recorded with a digital camera (E-450, Olympus, Tokyo, Japan). The color change of the freshness indicator was evaluated while it remained attached to the beef packaging together with the beef sample. Measurements were conducted in triplicate, and for each replicate, the color was recorded at a single randomly chosen point on the indicator surface. Colorimetric measurements were performed using a colorimeter (CR-300, Minolta, Tokyo, Japan). The color difference (ΔE) was calculated using Equation (2):

(2) $$\Delta E=\sqrt{{\left(L-L_{0}\right)}^{2}+{\left(a-a_{0}\right)}^{2}{\;+\;\left(b-b_{0}\right)}^{2}}$$

where ΔE is the color difference; *L*, *a*, and *b* represent darkness to lightness (0 to 100), the red/green coordinate, and the yellow/blue coordinate, respectively, of the freshness indicator after reaction with TMA; *L*_0_, *a*_0_, and *b*_0_ are the color values of the freshness indicator before reaction. The value of ΔE can be used as a scale to indicate whether a person can distinguish two colors [24]: ΔE < 3: no perceptible differences; 3 < ΔE < 6: very small differences; 6 < ΔE < 9: fairly perceptible differences; 9 < ΔE < 12: perceptible differences; ΔE > 12: different colors.

### 2.5. Beef Quality Assessment

Beef quality was assessed by measuring the TBC, TVB-N, and pH value every 2 days.

#### 2.5.1. Microbial Analysis

Ten grams of stored beef was put into a stomacher bag containing 90 mL of peptone water and stomached for 2 min. One milliliter of beef juice was extracted and serially diluted using peptone water. Then, diluted juice was spread onto the Petrifilm™ Aerobic Count Plate (3M, Elyria, OH, USA) in duplicate. The plate was incubated at 37 °C for 48 h to determine the TBC.

#### 2.5.2. TVB-N Measurement

One hundred grams of stored beef loin was well minced, and 5 g of minced beef was placed in a centrifuge tube. Next, 25 mL of double-distilled water was added to the centrifuge tube and homogenized at 7000 rpm for 30 s. The suspension was filtered. One milliliter each of the filtrate and supersaturated potassium solution was dispensed into the outer chamber of a Conway microdiffusion cell, and 1 mL of 0.01 N sulfuric acid was dispensed into the inner chamber of the cell. The cell was incubated at 20 °C for 1 h. The inner chamber solution (1 mL of 0.01 N sulfuric acid) was then titrated with 0.01 N sodium hydroxide.

TVB-N was calculated using Equation (3):

(3) $$TVB-N\;\left(\frac{mg}{100}g\right)=0.14\;\times \frac{\left(V-V_{0}\right)}{W}\;\times \;100\;\times \;d$$

where *V* is the volume of sodium hydroxide used for titrating the test sample, *V*_0_ is the volume of sodium hydroxide used for titrating the blank sample, *W* is the weight of the beef loin sample, and *d* is a dilution factor.

#### 2.5.3. pH Measurement

The pH of the filtrate (see Section 2.5.2) was measured by a pH meter (Orion Star A211 Conductivity Benchtop Meter, Thermo-Fisher Scientific, Waltham, MA, USA).

### 2.6. Headspace Analysis of Beef Packaging

The headspace of the beef package was analyzed using a gas chromatograph (GC 7890A, Agilent Technologies, Santa Clara, CA, USA) coupled with a flame ionization detector after SPME. The extraction was performed using a 75 µm CAR/PDMS fiber to analyze volatile amines, including MA, DMA, and TMA. Quantification was conducted using the external standard method. Individual 1000 μg/mL stock solutions of three amines were prepared. Working solutions were prepared by diluting the stock solutions with double-distilled water. One milliliter of amine solution was placed into the 22 mL vial and hermetically sealed with a PTFE septum. Then, 1 mL of 50% sodium hydroxide was injected into the vial using a syringe and vortexed for 1 min to salt out the volatile amines [25,26]. The concentration of vaporized amines was 1, 2, 5, 10, and 20 μg/kg.

To analyze the package’s headspace, a septum was attached to the packaging film using instant glue (Loctite, Henkel, Belgium), and the packaging was left for 48 h to allow the glue to solidify [27]. Then, the SPME fiber was pierced through the septum and exposed to the headspace of the package for 30 min. After extraction, desorption of volatile compounds was carried out in the injection port for 5 min in splitless mode. The temperature of the injection port was 250 °C. A glass liner (0.7 mm ID) was equipped. A CP-Volamine 7447 capillary column (30 m × 0.32 mm ID; Agilent Technologies, Santa Clara, CA, USA) was used for volatile amine separation. Air and hydrogen were supplied at flow rates of 40 and 400 mL/min, respectively. The oven temperature was set at 40 °C for 5 min, followed by an 11 °C/min ramp to 120 °C, then a 35 °C/min ramp to 250 °C, and held at 250 °C for 5 min. The detector temperature was 250 °C.

### 2.7. Standardized Kinetic Framework for Cross-Indicator Comparison

We compared indicators that have different units and ranges using a single, standardized procedure. First, each time series was scaled to [0, 1] using min–max normalization. We then fitted a two-parameter logistic model with the upper asymptote fixed at 1 (4):

(4) $$Y_{norm}(t)=\frac{1}{1+exp[-k(t-t_{0})]}$$

where *k* denotes the steepness, and *t*_0_ is the time at which the rise is centered. Parameters *k* and *t*_0_ were estimated by nonlinear least squares on the normalized data. Model performance was assessed on the same normalized scale using root mean square error (RMSE) and *R*^2^, and the maximum instantaneous slope was summarized as *v*_max_ = *k*/4. Values reported as non-detects were treated as missing in the primary analysis.

This standardized logistic fit was the only method used to enable fair, unit-agnostic comparisons of speed (*k*) and timing (*t*_0_) across indicators. Practical decision thresholds (e.g., ΔE = 12, TBC = 7 log CFU g^−1^, TVB-N = 20 mg/100 g^−1^) are referenced elsewhere for interpretation, but they were not used to fit additional models nor to derive alternative timing estimates within this section.

### 2.8. Pearson’s Correlation Method

A correlation analysis was performed to examine the relationship between the color difference (ΔE*) of the freshness indicator and the concentration of volatile amines (MA, DMA, and TMA) in the package headspace. The Pearson’s correlation coefficient (r) was calculated using the following Equation (5):

(5) $$r=\frac{\sum_{i=1}^{n}\left(x_{i}-\overset{-}{x})(y_{i}-\overset{-}{y}\right)}{\sqrt{\sum_{i=1}^{n}(x_{i}-\overset{-}{x}{)}^{2}\sum_{i=1}^{n}(y_{i}-\overset{-}{y}{)}^{2}}}$$

where $x_{i}$ and $y_{i}$ represent the paired measurements of amine concentration (µg/kg) and ΔE, respectively, and $\overset{-}{x}$ and $\overset{-}{y}$ denote their mean values. The value of *r* ranges from −1 to +1, where values closer to +1 indicate a strong positive correlation, values near −1 indicate a strong negative correlation, and values around 0 indicate no linear relationship. The statistical significance of the correlation was evaluated at a confidence level of *p* < 0.05. All calculations were performed using SPSS Statistics 26.0 software (IBM Corp., Armonk, NY, USA).

### 2.9. Statistical Analysis

All experiments were performed in triplicate, and the results were expressed as mean ± standard deviation (SD). The coefficient of variation (CV, %) was additionally calculated as (SD/mean) × 100 to evaluate the dispersion and reproducibility of replicate measurements. Statistical analyses were performed using SPSS Statistics 26.0 software (IBM Corp., Armonk, NY, USA). One-way analysis of variance (ANOVA) followed by Tukey’s post hoc test was applied to determine significant differences among sample groups, with a confidence level of *p* < 0.05. The relationship between the ΔE of the indicator film and freshness parameters such as TVB-N, pH, and TBC was analyzed using the Pearson correlation model. The correlation coefficients (*r*) were interpreted according to their direction and magnitude to evaluate the consistency of the observed trends. All statistical evaluations were based on independent experimental replicates obtained under identical conditions.

## 3. Results and Discussion

### 3.1. Optimization and Colorimetric Response Under Simulated Volatile Amines

The numbers (20, 60, 100, and 140) indicate plasticizer content (% *w*/*w* vs. CA), and the suffixes -1/-2 denote dye ratios of 1:1 and 1:2. The visual color difference threshold was set to ΔE = 12 based on prior literature and our internal standard [28,29,30]. This ΔE = 12 threshold matches the grading table in Section 2. At the screening stage, we evaluated both the first TMA concentration at which ΔE = 12 is reached and the final contrast at 50 μg/mL to compare early sensitivity and late-stage visibility [30].

As TMA increased from 0 to 50 μg/mL, ΔE rose monotonically for all films, and at 0 μg/mL of TMA, ΔE was ≈ 0, confirming baseline stability (Table 2). At 10 μg/mL of TMA (early sensitivity), ΔE values were generally higher for the BCG:BCP = 1:1 group than the 1:2 groups. For example, 400P60-1 showed the highest value (19.28), followed by 200P60-1 (17.73), 400P100-1 (17.69), 200P100-1 (16.79), G140-1 (15.99), and G60-1 (15.89). Several glycerol-based 1:2 formulations showed lower ΔE than the 1:1 formulations at the same concentration.

Threshold reactivity was assessed as the first TMA concentration at which ΔE = 12 is reached. Many 1:1 formulations already met the threshold at 10 μg/mL, including G100-1, G140-1, 200P20-1, 200P60-1, 400P60-1, and 400P100-1. In contrast, some 1:2 formulations required higher concentrations (for example, 20–50 μg/mL). Notably, 200P100-2 did not reach ΔE = 12, even at 50 μg/mL.

At 50 μg/mL (final contrast), G100-1 showed the largest ΔE (47.80), followed by G140-1 (45.96), 200P20-1 (45.70), and 400P60-1 (44.18). Additionally, 400P20-1 (39.60) and 200P60-1 (39.58) also gave sufficient contrast. Some 1:2 formulations showed lower final ΔE values than the 1:1 formulations, for example, 200P100-2 (18.43), 400P100-2 (17.02), and 200P140-2 (10.19).

**Figure 2:**
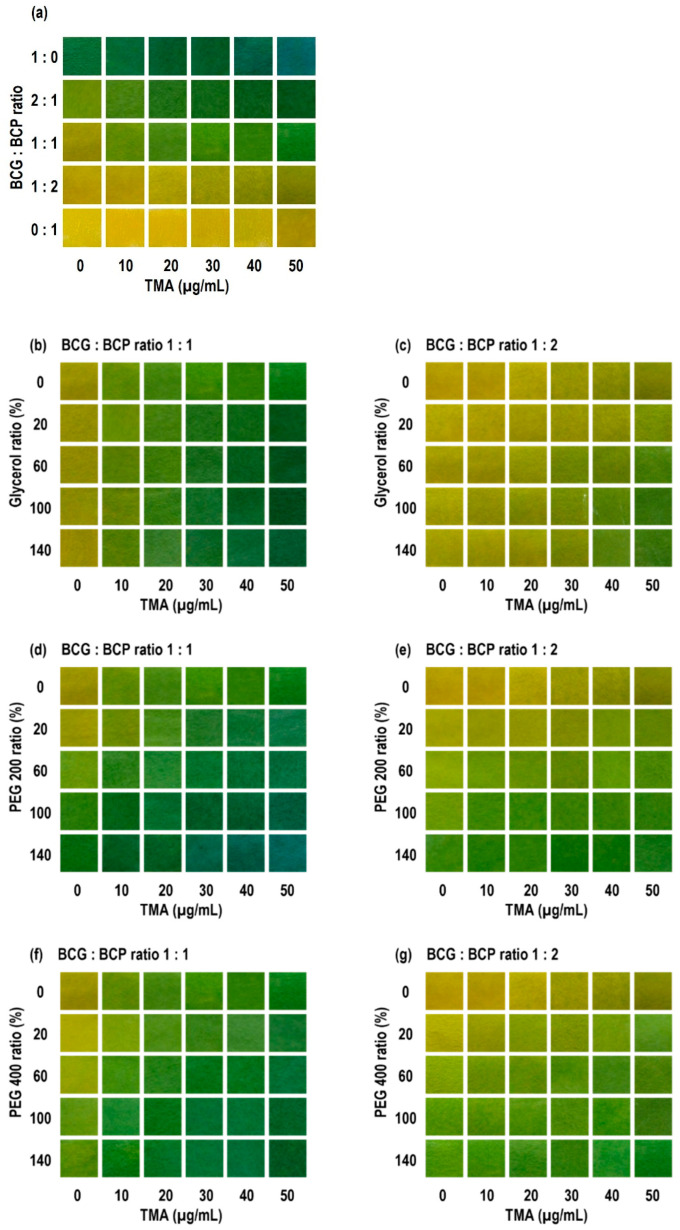
Freshness indicator exposed to TMA at different concentrations (0, 10, 20, 30, 40, 50 µg/mL). (**a**) Ratio of BCG and BCP (1:0, 2:1, 1:1, 1:2, 0:1) in the G100 formulation; (**b**,**c**) freshness indicator with glycerol in different ratio of 20, 40, 100, 140% to CA; (**d**,**e**) freshness indicator with PEG200 in different ratio of 20, 40, 100, 140% to CA; (**f**,**g**) freshness indicator with PEG400 in different ratio of 20, 40, 100, 140% to CA.

Figure 2 visually supports these quantitative results. All formulations exhibited a gradual, stepwise color transition as the pH changed. They appeared yellow in the acid form, shifted to a yellow-green shade at intermediate conditions, and finally turned green in the base form. Mixed dyes showed a sharper transition boundary than single dyes. G100-1 and G100-2 maintained a stable yellow baseline at 0 μg/mL of TMA, ensuring a higher sensitivity at low concentrations. As concentration increased, brightness (*L*) decreased, and the green component increased uniformly across tiles. Glycerol systems kept a yellow baseline, giving a wide transition window. Some PEG systems showed a green tone even without exposure, which could limit the extra color change at the start. Nonetheless, several PEG200/PEG400 middle- to high-loading films turned uniformly green as concentration increased, which improved readability in the higher range. In terms of spatial uniformity, glycerol systems showed smaller tile-to-tile variation in color difference, while a few high-PEG films showed early edge coloration. All images were taken and analyzed under identical camera and lighting conditions, and white balance effects were controlled as described in the Methods.

The key finding from this screening is that dye ratio, plasticizer type, and loading systematically separate early sensitivity, threshold attainment, and late-stage contrast. For the dye ratio (BCG:BCP), BCG (effective pH ≈ 3.8–5.4) transforms to its base form at a lower pH than that of BCP (pH ≈ 5.2–6.8). A 1:1 ratio, therefore, tends to trigger earlier in the low-to-middle concentration range and acts as an “early-warning” type. A 1:2 ratio increases the contribution of BCP, resulting in color saturation loss and greening buildup in the middle-to-high range, which provides stronger late-stage contrast. This finding aligns with previous reports on the distinct pH ranges and the benefits of mixing the two indicators, and it supports our decision to prioritize 1:1 and 1:2 ratios in screening [31,32,33,34].

The effect of the plasticizer system on the transition was consistent across visual and quantitative readouts. Glycerol systems maintained a stable yellow baseline at 0 μg/mL of TMA, resulting in a wide transition window (headroom), and with increasing concentration of TMA, both *L* loss and green gain proceeded smoothly and together, giving high spatial uniformity. Some PEG200/PEG400 systems showed a green tone at zero exposure, which can reduce the extra color change at the start. However, in the middle-to-high range of TMA concentration, they showed panel-wide uniform greening that improves late-stage readability. These differences can be explained by the distinct effects of glycerol and PEG on plasticization, hydrogen-bond networks, and diffusion paths, which is consistent with observations in materials used in food packaging films and sensors [35,36].

The choice and use of the threshold also make the interpretation robust. Using the ΔE grading (refer to the Methods), many 1:1 formulations already showed ΔE > 12 at 10 μg/mL of TMA and could be visually read as a “different color.” Some 1:2 formulations reached the threshold only at higher concentrations, although their final ΔE at 50 μg/mL still provided sufficient contrast. In other words, using the same threshold (ΔE = 12), the time or concentration at which it is reached separates operational behavior. This threshold classification can be reused later in actual package tests as a consistent, non-destructive reading rule linked to quality metrics [37].

Spatial uniformity and reproducibility matter for field use. G100-1 and G100-2 changed uniformly across tiles with little banding or blotches. Under the same imaging conditions and controlled white balance, between-observer variation remained small. In a few high-PEG films, early edge coloration suggests effects from label position and headspace airflow during packaging. This aligns with reports recommending that pH-responsive labels be placed inside the headspace and close to the sample to reduce gas distribution effects [38]. The relatively high plasticizer-to-CA ratios (100%, *w*/*w*) used in this study did not compromise film integrity. These formulations remained mechanically stable and were intentionally designed to fine-tune the indicator’s response kinetics to match the gradual spoilage of beef. Section 3.3 addresses this with standardized label placement and a consistent headspace layer.

From a combined quantitative and qualitative perspective, Table 2 and Figure 2 show the same phenomenon at different resolutions. For example, G100-1 exceeds the threshold (ΔE > 12) already at 10 μg/mL and reaches a maximum ΔE of ≈ 47.8 at 50 μg/mL, thereby securing both early warning and final visibility. G100-2 is more conservative at low levels but adds contrast steadily in the middle-to-high range, which supports readability later on. This trade-off between transition speed and final contrast also appears during storage. Early in storage, G100-1 gives a larger ΔE, then around day 4, the curves cross, and G100-2 becomes larger. The most likely reason is that the two indicators sample different parts of the same amine buildup curve because their pH response ranges are different.

### 3.2. Application of Freshness Indicators to Beef Packaging During Storage

During refrigerated storage (0–10 days), both microbiological and chemical quality markers of packaged beef underwent marked changes (Table 3). TBC gradually increased over the period: 3.00 log CFU/g on day 0, 4.51 on day 2, 5.92 on day 4, 6.92 on day 6, 8.36 on day 8, and 8.64 on day 10. By linear interpolation, the time to reach 7 log CFU/g was about 6.11 days. A threshold of 7 log CFU/g is widely used in fresh meat as a practical spoilage limit [39,40]. TVB-N showed a continuous increase throughout storage. The value was 7.00 ± 0.00 mg/100 g on day 0, 10.20 ± 0.80 on day 2, 14.00 ± 0.00 on day 4, 14.40 ± 0.80 on day 6, 18.20 ± 1.40 on day 8, and 23.80 ± 1.40 mg/100 g on day 10. Linear interpolation between days 8 and 10 indicated that 20 mg/100 g was reached at approximately 8.64 days. TVB-N reflects the accumulation of volatile amines and ammonia during storage [41,42], and around 20 mg/100 g is often adopted as a practical spoilage threshold for fresh meat [43,44]. The pH increased modestly from 5.72 ± 0.02 (day 0) to 6.24 ± 0.08 (day 10) (day 2: 5.86 ± 0.01; day 4: 5.92 ± 0.01; day 6: 5.96 ± 0.01; day 8: 6.05 ± 0.17). This slow increase is typical for chilled meat, where early buffering is followed by a later accumulation of basic metabolites [45].

Under these storage conditions, the microbiological limit was reached about 2.5 days earlier than the chemical limit. This ordering matches prior observations that microbiological change tends to lead while TVB-N lags, and it is consistent with the known sequence of spoilage reactions that ultimately generate volatile bases [41]. The mechanistic picture also aligns with reports of delayed amine build-up in the package headspace during refrigerated storage [46,47].

For application, the alarm threshold should be positioned so that the visible color change occurs by roughly 6.1 days, which provides usable lead time. Recent work shows that ΔE correlates significantly with TBC and is also associated with TVB-N, supporting the use of a colorimetric label as a practical quality indicator in real packaging environments [42,45]. In practice, the time to ΔE ≥ 12 should be earlier than, or at least close to, the 7 log CFU/g point to enable timely intervention; if ΔE appears only after TVB-N surpasses 20 mg/100 g, the alert is late and the threshold or label chemistry should be adjusted.

With this requirement in mind, our screening indicates that glycerol-based 1:1 films are the most reliable choices for early warning. In particular, G100-1 and G140-1 exceed ΔE ≥ 12 at low exposure (10 μg/mL gives 12.17 and 15.99, respectively), maintain a stable yellow baseline at 0 μg/mL, and provide high late-stage contrast at 50 μg/mL (47.80 and 45.96), with good spatial uniformity. When PEG matrices are required, 400P60-1 and 200P20-1 also trigger early at 10 μg/mL (19.28 and 13.97) and deliver adequate final contrast at 50 μg/mL (44.18 and 45.70). Because some PEG films can show a slight green tone even without exposure, the baseline hue should be verified in packages to avoid premature greening. Most 1:2 formulations trigger later and show lower final contrast, so they are less suitable for an early-warning target. These choices are consistent with studies that treat 7 log CFU/g as a working microbiological criterion for chilled beef and that recognize about 20 mg/100 g TVB-N as a practical chemical limit in industry settings [48,49,50].

### 3.3. Headspace Volatile Amines Analysis

**Figure 3:**
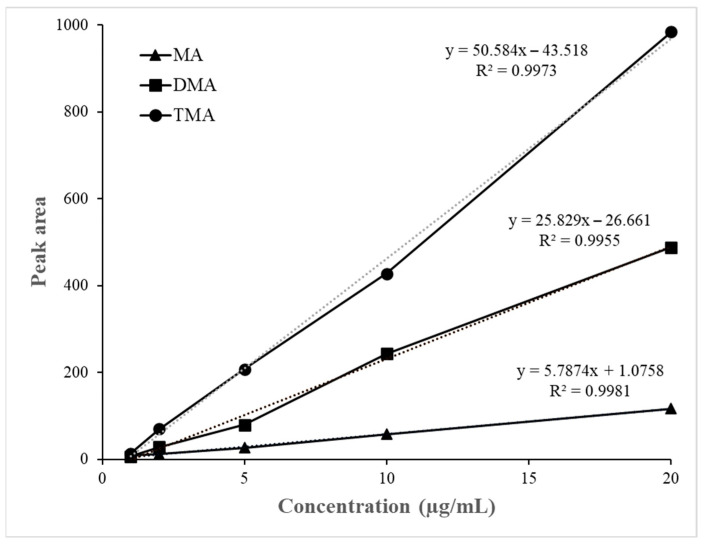
Calibration curves of MA, DMA and TMA.

The calibration curves for MA, DMA, and TMA showed excellent linearity within the tested range (0–20 µg/mL), with correlation coefficients (R^2^) of 0.9981, 0.9955, and 0.9973, respectively (Figure 3).

During chilled storage, the concentration of volatile amines in the package’s headspace increased over time (Table 4). From day 2 onward, DMA was present at low levels and increased steadily: 2.28 ± 0.12 µg/kg (day 2), 2.35 ± 0.18 µg/kg (day 4), 2.83 ± 0.15 µg/kg (day 6), 4.87 ± 0.27 µg/kg (day 8), and 7.37 ± 0.32 µg/kg (day 10). MA was below the detection limit from days 2 to 6, then rose to 3.19 ± 0.21 µg/kg on day 8 and 6.96 ± 0.34 µg/kg on day 10. TMA likewise shifted from non-detectable on days 2–6 to 2.03 ± 0.14 µg/kg on day 8 and 3.29 ± 0.22 µg/kg on day 10. Total amines reached 10.09 µg/kg on day 8 (DMA 48.3%, MA 31.6%, TMA 20.1%) and 17.62 µg/kg on day 10 (DMA 41.8%, MA 39.5%, TMA 18.7%). All replicate measurements showed low coefficients of variation (CV ≤ 8%), confirming the reproducibility and consistency of the analytical results. To provide quantitative context for these levels, previous studies have reported that in chilled meat systems MA typically reaches approximately 3–4 µg/g, DMA ranges from 0.25 to 11 µg/g, and TMA can increase from 0.44 µg/g to nearly 39 µg/g during early spoilage, with off-flavor becoming noticeable at around 25 µg/g [21]. Although the absolute TMA values reported were obtained under higher-temperature storage conditions—and therefore exceed those observed in our chilled system—the relative order and buildup pattern of MA, DMA, and TMA remain representative of early spoilage progression [21]. Taken together, these magnitudes correspond well to the concentration range at which our labels first exceeded ΔE ≈ 12, supporting the relevance of this threshold as an early-warning indicator. The stronger late increase is consistent with the TVB-N buildup pattern typically observed in chilled meats [41].

The headspace profile showed a two-stage rise: DMA appeared first, followed by a sharper late increase in MA and TMA. This is consistent with the established route in which bacterial growth is followed by protein and amino-acid breakdown and subsequent deamination yielding volatile bases [51]. Moreover, beef generally contains less of the TMA precursor TMAO than seafood, so TMA tends to appear later and remain lower in absolute amount; our data replicate this timing, with TMA non-detectable until day 8 and increasing only toward the end [52].

The timing also aligns with quality limits derived for chilled beef. TBC reached 7 log CFU/g at about 6.11 days by linear interpolation, whereas TVB-N reached 20 mg/100 g at about 8.64 days, indicating that the microbiological limit precedes the chemical markers and the headspace amines [43]. Given this order, the visible threshold of the color label (e.g., ΔE ≥ 12) should be reached around day 6 under the same storage conditions to ensure a practical lead time; during days 8–10, the slopes were ≈ +1.89 µg/kg·day^−1^ for MA, +1.25 µg/kg·day^−1^ for DMA, and +0.63 µg/kg·day^−1^ for TMA, so MA drives the late rise in the total basic load. To maintain reliable late-phase readability, the label should be tuned so that contrast continues to build as the basic load increases [45].

A practical limitation of this study is that indicator screening was performed in model TMA atmospheres, whereas the beef headspace becomes DMA-rich in the later storage period. This discrepancy does not undermine the approach, because pH-sensitive indicator films respond to the total basic load (TVB-N) rather than to a single amine species, and mixed-amine responses, including partial discrimination among MA, DMA, and TMA, have been reported for colorimetric systems [53,54]. Consistent with this, BCG-based films are broadly sensitive to low-molecular-weight basic gases [55], and under model TMA they often exhibit exponential or logistic color-change trajectories with concentration, a behavior that mechanistically allows faster ΔE buildup in mixed-amine atmospheres. In our package tests, ΔE was aligned with microbiological and chemical endpoints (7 log CFU/g and approximately 20 mg/100 g TVB-N), which supports operational validity even when DMA predominates [50,52].

The concentration of volatile amines in the headspace was calculated using Equation (1), which relates the measured GC–FID peak area to the gas-phase concentration under ideal assumptions. It should be noted, however, that this calculation is dimensionally consistent but neglects gas-phase thermodynamic activity and possible wall-adsorption losses. Because such losses can occur during sampling and equilibration, the actual influence of TMA, DMA, and MA on the headspace composition is likely greater than the nominal values reported. Nevertheless, these limitations do not materially affect the overall trend or the interpretation of the colorimetric response, which remains robust across replicates and storage periods.

### 3.4. Pearson’s Correlation Between Label ΔE* and Headspace Amines

To evaluate the linear relationship between the color variation (ΔE) of the indicator label and the concentrations of headspace amines (MA, DMA, and TMA), the Pearson correlation model—the simplest and most widely used method for assessing linear association—was applied. Because the number of data points per condition was limited (n = 3), the statistical confidence of the correlation coefficients may be reduced; therefore, the results are interpreted primarily as indicative of consistent trends rather than as statistically predictive values.

**Figure 4:**
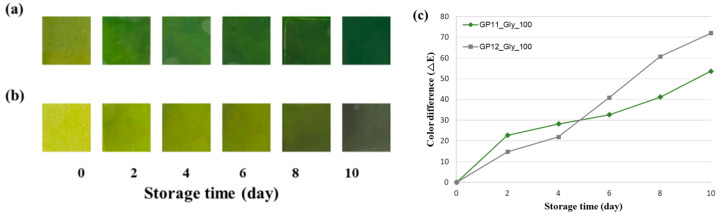
Freshness indicator during storage (**a**) GP11_Gly_100 (**b**) GP12_Gly_100, (**c**) color difference of freshness indicators during storage.

Figure 4a,b illustrate the progressive color change of the indicator labels (G100-1 and G100-2) during 10 days of chilled storage. The color gradually shifted from yellow-green to dark green or gray, reflecting the accumulation of volatile amines and the onset of spoilage. Correspondingly, the quantified color difference (ΔE) increased continuously with storage time (Figure 4c).

As summarized in Table 5, ΔE exhibited strong positive correlations with both DMA and total volatile amine concentrations. For G100-1, the ΔE–DMA correlation showed r = 0.956, *p* = 0.003, and the ΔE–total amines correlation showed r = 0.945, *p* = 0.004, both indicating strong positive and significant correlations. G100-2 showed a consistent pattern of correlations similar to that observed for G100-1, with ΔE–DMA r = 0.959, *p* = 0.002, and ΔE–total amines r = 0.936, *p* = 0.006. Similar degrees of label–quality correlation (ΔE with TVB-N or microbial counts) have been reported repeatedly in packaged fresh meat, especially beef [56,57,58].

Similar mechanistic behavior has been reported for polymer-film amine sensors, where the response kinetics are governed by the gas–solid partition coefficient of each amine and rapid acid–base or coordination equilibrium within the film. Because low-molecular-weight amines can undergo partial adsorption onto polymer surfaces or container walls, the effective gas-phase activity may deviate from the nominal concentration introduced into the system [59]. These adsorption effects generally reduce absolute headspace values but do not alter the monotonic buildup of volatile bases during spoilage. Accordingly, the ΔE trajectory in our system is primarily driven by the accumulation of total basic load in the headspace rather than by limitations in dye-equilibration kinetics, which mechanistically supports the observed sigmoidal pattern.

The two labels demonstrated nearly identical correlation strengths, indicating that their color responses were consistently driven by the same underlying chemical factor, namely the accumulation of volatile amines. Although the two systems differ in their dye composition ratio, this difference did not significantly affect the sensitivity or stability of their colorimetric responses. From a functional standpoint, G100-1 and G100-2 can serve complementary purposes in monitoring freshness. The stronger ΔE–DMA correlation observed in G100-1 supports its use for early detection, providing a sufficient lead time before the microbial limit of 7 log CFU/g is reached (approximately six days). In contrast, the stronger ΔE*–total amine correlation in G100-2 suggests its suitability for late-stage assessment, particularly near the TVB-N threshold of 20 mg/100 g (around nine days). Since beef contains relatively low levels of the TMA precursor, TMAO, the formation of TMA occurs later and at lower concentrations. Consequently, total volatile amines become a more reliable indicator of freshness in the final phase of storage, which is consistent with the results observed in this study [52,60].

From a practical standpoint, these relationships provide a basis for threshold-based decision rules. For example, if ΔE ≥ 12 is used as an early-warning criterion, the robust ΔE–DMA correlation ensures sufficient lead-time for quality control. Conversely, the ΔE–total amine correlation justifies selecting a label formulation such as G100-2 for end-of-life indication. Previous studies have likewise reported that pH-sensitive films provide quantitative color responses to mixed amine vapors, consistent with the correlation structure observed herein [7].

### 3.5. Linkage Between ΔE Thresholds and Spoilage Factors with Kinetics

The standardized fits in Table 6 allow us to compare the “speed” and “timing” of ΔE curves and quality indicators on the same scale. In the normalized logistic model, the slope (*k*) captures how steeply the rise occurs, and the center time, *t*_0_, marks when the rise becomes pronounced [61]. In our data, k values were 0.601 for TBC, 0.583 for ΔE (G100-2), 0.447 for TVB-N, and 0.405 for ΔE (G100-1). The corresponding maximum instantaneous slope (*V_max_* = *k*/4) was therefore higher for TBC (≈0.150 day^−1^) and ΔE (G100-2) (≈0.146 day^−1^) than for TVB-N (≈0.112 day^−1^) and ΔE (G100-1) (≈0.101 day^−1^). In other words, once the increase begins, TBC and ΔE (G100-2) accumulate faster, while ΔE (G100-1) and TVB-N progress more gradually [62].

The timing summary tells a complementary story. The earliest t0 was for TBC at 4.065 days, with ΔE (G100-1) essentially overlapping at 4.11 days. ΔE (G100-2) followed at 5.21 days and TVB-N at 5.73 days. Taken together, these parameters simultaneously characterize the order in which quality indicators initiate and the rate at which their changes proceed once initiated. Comparing the two labels, ΔE (G100-1) starts early but is less steep, which fits an early-transition profile, and ΔE (G100-2) starts later but climbs quickly after the transition, which fits a late-acceleration profile [58].

Model performance on the normalized scale was very good. For ΔE (G100-2) and TBC, respectively, R^2^ was 0.981 and 0.980 with RMSE 0.034 and 0.043, showing that the model explains the changes almost completely. TVB-N showed R^2^ = 0.927, RMSE = 0.062, which is sound, and ΔE (G100-1) showed R^2^ = 0.892, RMSE = 0.074, which is still adequate. This means we can reliably compare the kinetic positions of the two ΔE labels against indicators that have different units and ranges. This normalized logistic frame is consistent with how first-order models are commonly interpreted for predicting the storage life of fresh meat, as well as meat spoilage, including logistic, Gompertz, and Baranyi forms [61,62].

Linking these kinetics to threshold times provides a clear operational picture. The near-match in t0 between TBC and ΔE (G100-1) implies that, under the same storage conditions, the ΔE threshold used for visual reading in this study (ΔE ≥ 12) can align closely on the time axis with the microbiological limit (TBC = 7 log CFU/g) [63,64]. In Section 3.2, the estimated time to reach 7 log CFU/g was about 6.1 days. Because ΔE (G100-1) transitions earlier than ΔE (G100-2), it is likely to reach ΔE ≥ 12 before or at least near that point, which supports an early-warning role. By contrast, ΔE (G100-2) has a later *t*_0_ (5.2 days) and a larger k, so it steepens quickly in the late phase. This aligns better with the TVB-N decision zone around 20 mg/100 g, which we estimated at approximately 8.6 days [35,39]. In short, ΔE (G100-1) is well placed to secure lead time relative to the microbiological limit, while ΔE (G100-2) is well placed to maintain strong contrast and near-concurrent signaling around the chemical limit. This finding aligns with prior work demonstrating that ΔE is correlated with TVC and TVB-N, and that label kinetics can track spoilage-indicator kinetics [65].

If early cutoff is the priority, it is reasonable to use ΔE (G100-1) as the primary label and fine-tune the label composition, thickness, and plasticizer so that ΔE ≥ 12 occurs before TBC reaches 7 log CFU/g. If reliable late-stage reading is more important, running ΔE (G100-2) in parallel helps build sufficient contrast as the end of shelf-life approaches. The high R2 and low RMSE in our standardized fits show that both labels stably track the main trend and timing of spoilage. Therefore, the ΔE threshold should be treated not as an isolated metric but as an operating criterion linked to the kinetics of TBC and TVB-N.

## 4. Conclusions

This study, from an analytical chemistry perspective, demonstrated that the ΔE-based color difference indicator reliably reflects microbiological quality and volatile amine accumulation during refrigerated storage of beef. Fitting ΔE time series to a normalized logistic model allowed the extraction of key kinetic parameters, namely the slope (*k*) and center time (*t*_0_), thereby linking the indicator response with both the rate and timing of spoilage. TBC and ΔE (G100-2) exhibited steeper increases during the acceleration phase, while TVB-N and ΔE (G100-1) increased more gradually, confirming that the indicator encodes complementary aspects of quality deterioration.

From an operational perspective, a ΔE threshold of 12 was identified as a practical compromise that balances consumer readability with microbiological safety. Importantly, the division of roles between the two formulations was clear: G100-1 provided a rapid early-warning signal, whereas G100-2 maintained strong contrast in later storage stages, thereby enhancing end-of-life readability. This dual-label configuration therefore supports a robust and intuitive decision-making framework for both quality control checkpoints and consumer use.

Overall, the proposed ΔE-threshold-based indicator system, combined with a standardized kinetic interpretation framework, establishes a practical and scientifically grounded quality management tool. By covering both early detection and late-stage readability, this approach can improve safety assurance and communication in the refrigerated beef supply chain. Future work should validate consumer acceptance of the dual-label design and explore its applicability across diverse food matrices and storage conditions.

## Figures and Tables

**Table 1:** Sample codes and compositions of cellulose acetate indicator films prepared with BCG/BCP and plasticizers.

Code	Matrix (Polymer/Substrate/Lamination)	BCG:BCP Ratio	Plasticizer Type	Initial Color at 0 µg/mL TMA	Plasticizer Conc. (% of CA)
G20-1	CA-coated filter paper PTFE-D support PET lamination	1:1	Glycerol	Yellow (acidic form) at 0 µg/mL TMA	20
G60-1					60
G100-1					100
G140-1					140
200P20-1			PEG200	Green (basic form) at 0 µg/mL TMA	20
200P60-1					60
200P100-1					100
200P140-1					140
400P20-1			PEG400		20
400P60-1					60
400P100-1					100
400P140-1					140
G20-2		1:2	Glycerol	Yellow (acidic form) at 0 µg/mL TMA	20
G60-2					60
G100-2					100
G140-2					140
200P20-2			PEG200	Green (basic form) at 0 µg/mL TMA	20
200P60-2					60
200P100-2					100
200P140-2					140
400P20-2			PEG400		20
400P60-2					60
400P100-2					100
400P140-2					140

**Table 2:** Color difference (ΔE) responses of cellulose acetate indicator films at different TMA concentrations.

Freshness Indicator	TMA Concentration (µg/mL)					
	0 µg/mL	10 µg/mL	20 µg/mL	30 µg/mL	40 µg/mL	50 µg/mL
	ΔE					
G20-1	0	13.87 ± 0.16	21.63 ± 0.09	21.63 ± 0.27	32.36 ± 0.18	34.53 ± 0.65
G60-1	0	15.89 ± 1.49	21.43 ± 0.63	34.61 ± 0.94	39.61 ± 0.74	39.61 ± 0.34
G100-1	0	12.17 ± 0.51	25.31 ± 0.05	35.00 ± 0.63	39.58 ± 1.21	47.80 ± 0.18
G140-1	0	15.99 ± 1.77	27.96 ± 1.20	36.56 ± 0.65	41.38 ± 0.73	45.96 ± 2.23
200P20-1	0	13.97 ± 1.19	26.60 ± 1.17	38.15 ± 0.80	42.80 ± 0.89	45.70 ± 2.48
200P60-1	0	17.73 ± 0.28	22.39 ± 0.29	30.18 ± 0.07	30.18 ± 0.21	39.58 ± 0.04
200P100-1	0	16.79 ± 0.66	18.96 ± 0.54	25.53 ± 0.57	29.00 ± 0.73	35.21 ± 0.36
200P140-1	0	9.84 ± 0.18	9.33 ± 0.41	23.29 ± 0.20	26.48 ± 0.25	40.97 ± 0.48
400P20-1	0	14.11 ± 0.74	26.91 ± 0.46	33.01 ± 0.63	35.72 ± 0.79	39.60 ± 1.26
400P60-1	0	19.28 ± 0.35	27.60 ± 0.46	35.79 ± 0.44	36.79 ± 0.12	44.18 ± 1.43
400P100-1	0	17.69 ± 1.00	21.07 ± 1.62	28.95 ± 0.20	31.55 ± 0.48	35.77 ± 0.14
400P140-1	0	15.74 ± 0.78	26.64 ± 0.20	29.39 ± 0.29	32.36 ± 0.51	38.12 ± 0.44
G20-2	0	1.40 ± 0.79	5.24 ± 0.44	9.46 ± 0.90	11.18 ± 0.66	18.86 ± 0.21
G60-2	0	1.66 ± 0.37	8.81 ± 0.63	16.68 ± 1.23	20.48 ± 0.76	26.19 ± 0.73
G100-2	0	7.68 ± 0.37	16.45 ± 0.48	18.57 ± 2.44	27.27 ± 0.28	33.03 ± 0.86
G140-2	0	5.34 ± 1.75	10.42 ± 0.28	16.34 ± 0.04	16.34 ± 0.58	31.30 ± 0.61
200P20-2	0	5.23 ± 0.22	5.27 ± 1.59	8.92 ± 0.99	14.03 ± 0.86	18.88 ± 0.86
200P60-2	0	9.50 ± 0.09	13.90 ± 0.57	14.96 ± 0.29	13.98 ± 0.41	19.82 ± 0.75
200P100-2	0	14.92 ± 0.32	16.05 ± 0.72	16.05 ± 0.27	18.93 ± 1.32	18.43 ± 0.18
200P140-2	0	3.15 ± 0.46	2.92 ± 0.30	8.67 ± 0.51	10.19 ± 0.44	10.19 ± 1.61
400P20-2	0	11.12 ± 0.04	15.76 ± 0.45	19.23 ± 0.33	21.55 ± 0.30	31.30 ± 0.23
400P60-2	0	11.81 ± 0.24	14.27 ± 0.18	19.06 ± 1.01	24.15 ± 0.01	26.62 ± 0.05
400P100-2	0	4.39 ± 0.62	2.98 ± 0.05	5.84 ± 0.47	8.37 ± 2.38	17.02 ± 1.07
400P140-2	0	4.31 ± 0.27	10.64 ± 0.45	12.15 ± 0.21	14.15 ± 1.47	20.74 ± 0.50

**Table 3:** Evaluation of total bacterial count (TBC), total volatile basic-nitrogen (TVB-N), and pH of Beef during refrigerated storage.

Parameter	Storage Time (Day)					
	0 Day	2 Days	4 Days	6 Days	8 Days	10 Days
TBC (log CFU/g)	3	4.51	5.92	6.92	8.36	8.64
TVB-N (mg/100 g)	7.00 ± 0.00	10.20 ± 0.80	14.00 ± 0.00	14.40 ± 0.80	18.20 ± 1.40	23.80 ± 1.40
pH	5.72 ± 0.02	5.86 ± 0.01	5.92 ± 0.01	5.96 ± 0.01	6.05 ± 0.17	6.24 ± 0.08

**Table 4:** Evaluation of MA, DMA, and TMA concentrations (µg/kg) and their Variability (CV%) in beef during refrigerated storage.

Parameter	Storage Time (Day)					
	0 Day	2 Days	4 Days	6 Days	8 Days	10 Days
MA (µg/kg)	-	n.d. *	n.d.	n.d.	3.19 ± 0.21	6.96 ± 0.34
CV (%)	-	-	-	-	6.6	4.9
DMA (µg/kg)	-	2.28 ± 0.12	2.35 ± 0.18	2.83 ± 0.15	4.87 ± 0.27	7.37 ± 0.32
CV (%)		5.3	7.7	5.3	5.5	4.3
TMA (µg/kg)	-	n.d.	n.d.	n.d.	2.03 ± 0.14	3.29 ± 0.22
CV (%)	-	-	-	-	6.9	6.7

* n.d.: not detected

**Table 5:** Evaluation of correlations between beef headspace volatile amines and freshness-indicator color difference (ΔE).

Freshness Indicator	DMA	Total Volatile Amines
GP11_Gly_100	0.956 *	0.945 *
	*p* = 0.003	*p* = 0.004
GP12_Gly_100	0.959 *	0.936 *
	*p* = 0.002	*p* = 0.006

* statiscally significant at *p* < 0.01.

**Table 6:** Standardized kinetic parameters across indicators.

Analyte	*k* (Normalized)	*t*_0_ (Day)	R^2^ (Norm)	RMSE (Norm)
ΔE (G100-1)	0.405	4.111	0.892	0.101
ΔE (G100-2)	0.583	5.211	0.981	0.049
TBC (log CFU/g)	0.601	4.065	0.98	0.051
TVB-N (mg/100 g)	0.447	5.726	0.927	0.087
DMA (μg/kg)	1.381	7.913	0.993	0.032

## Data Availability

The original contributions presented in this study are included in the article. Further inquiries can be directed to the corresponding author.
